# Urate Crystals; Beyond Joints

**DOI:** 10.3389/fmed.2021.649505

**Published:** 2021-06-04

**Authors:** Muhammad Israr Ahmad, Salman Masood, Daniel Moreira Furlanetto, Savvas Nicolaou

**Affiliations:** ^1^Department of Radiology, Faculty of Medicine, University of British Columbia, Vancouver, BC, Canada; ^2^Department of Radiology, Vancouver General Hospital, Vancouver, BC, Canada

**Keywords:** hyperuricemia, gout, DECT, extra-articular, MSU crystal deposition

## Abstract

Gout is the most common inflammatory arthropathy caused by the deposition of monosodium urate (MSU) crystals. The burden of gout is substantial with increasing prevalence of gout globally. The prevalence of Gout in the United States has increased by over 7% in the last two decades. Initially, it was believed that MSU crystal deposits occur only in the joints with the involvement of the periarticular soft tissues, but recent studies have shown the presence of MSU crystal deposition in extra-articular sites as well. Human plasma becomes supersaturated with uric acid at 6.8 mg/dl, a state called hyperuricemia. Beyond this level, uric acid crystals precipitate out of the plasma and deposit in soft tissues, joints, kidneys, etc. If left untreated, hyperuricemia leads to chronic gout characterized by the deposition of tophi in soft tissues such as the joints, tendons, and bursae. With the advent of newer imaging techniques such as DECT, MSU crystals can be visualized in various extra-articular sites. Extra-articular deposition of MSU crystals is believed to be the causative factor for the development of multiple comorbidities in gout patients. Here, we review the literature on extra-articular deposition of urate crystals and the role of dual-energy computed tomography (DECT) in elucidating multi-organ involvement. DECT has emerged as an invaluable alternative for accurate and efficient MSU crystal deposition detection. Future studies using DECT can help determine the clinical consequences of extra-articular deposition of MSU in gout patients.

## Introduction

Gout is the most common type of inflammatory arthritis characterized by the presence of monosodium urate (MSU) crystals in tissues. It is classified within the broader category of crystal deposition arthropathies. Additional entities within this category include calcium pyrophosphate dihydrate deposition disease, as well as hydroxyapatite deposition disease, which often manifest as the clinical syndromes of pseudo-gout and calcific tendinitis, respectively. The burden of gout is substantial, and epidemiological data suggest that the prevalence of gout may be increasing, with a global prevalence estimated at ~3% (1, 2). The United States has seen more than 7% increase in prevalence of gout over the past two decades (3). Some of the medical societies recommend that it should be treated with urate-lowering therapies only for the cases with acute form of disease (4).

Initially, it was believed that MSU crystal deposits occur only in the joints with involvement of the periarticular soft tissues, but many recent studies have shown the presence of MSU crystal deposition in extraarticular sites as well (5).

## Methodology

Articles related to the role of dual-energy computed tomography (DECT) in gout involving sites other than joints in English language were searched on PubMed and were included in this mini-review. The keywords that were searched included DECT, extraarticular gout and MSU deposition, cardiovascular involvement in gout, urate renal calculi, and DECT in gout with spinal involvement.

### Pathophysiology and Clinical Manifestations

Purines are building blocks of DNA and RNA present in cells of all living things and thus essential component of food. Purine metabolism leads to uric acid production, which is converted to allantoid in most mammals by uricase enzyme. However, in humans and higher apes, genes for the uricase enzyme are dysfunctional secondary to mutations, leading to high serum uric acid levels.

The human plasma becomes supersaturated with uric acid at 6.8 mg/dl, a state called hyperuricemia, which is common in general population. Beyond this level, uric acid crystals precipitate out of the plasma and deposit in soft tissues, joints, kidneys, etc.

Increased production, reduced renal excretion, or both are the basic mechanism causing hyperuricemia. It is influenced by other factors such as purine-rich diet, alcohol consumption, obesity, male gender, and use of diuretics. Hyperuricemia can be divided into primary and secondary types based on hereditary/idiopathic and acquired etiologies, respectively (6).

Several factors are thought to contribute to urate deposition in soft tissues. Low temperature is considered to be an important factor, as solubility of uric acid falls significantly with lower temperatures, leading to deposition in colder/peripheral parts of the body such as the pinna, olecranon tip, and distal interphalangeal joints of the fingers and toes. Other factors that influence urate deposition include level of cartilage hydration, local edema, fluctuating hyperuricemia, osteoarthritis, relative avascularity, and pH (7, 8).

Clinically, gout can present as rapid onset acute monoarticular/polyarticular disease in lower limb joints, most commonly the first MTP joint (podagra) (8). Symptoms include pain, fever, tenderness, swelling, and redness, and it may be difficult to distinguish acute gout from septic arthritis and cellulitis. The episodes are typically limited to 5–7 days.

If left untreated, hyperuricemia leads to chronic gout characterized by deposition of tophi in a variety of soft tissues including joint, tendons, and bursae (9). A tophus is a mass-like deposit of urate crystals and inflammatory cells along with calcium salts, fat, proteins, and polysaccharides (7).

Apart from peripheral articular involvement, gout can involve the axial skeleton. Rarely, it can involve temporomandibular joints as well (9). In an unusual presentation, gout may lead to cutaneous gouty panniculitis characterized by deposition of urate crystals in hypodermis (8).

Urinary tract stones and chronic urate nephropathy develop in up to 20% of patients. Gout not only causes urate stones in the kidneys but may also increase the risk of developing calcium oxlate stones.

There is a higher prevalence of cardiovascular and cerebrovascular diseases, hypertension, hypercholesterolemia, diabetes, renal disease, and obesity in gout patients compared to the controls. Andrés et al. (10) found that urate deposition in coronary arteries leads to worse coronary calcification and coronary artery disease. In an observational study, Park et al. (11) found histological evidence of urate crystal deposition in coronary arteries and prostate, which may lead to crystal-induced inflammation in these tissues.

Gout has a higher prevalence in men, postmenopausal women, and individuals with comorbidities such as cardiovascular disease, diabetes mellitus, chronic renal disease, and obesity (12).

### Association of MSU Crystal With Comorbidities

Extraarticular deposition of the MSU crystals is believed to be a causative factor for the development of multiple comorbidities in patients known to have gout. A survey done in 2007–2008 showed hypertension in 74% patients with gout, 71% had chronic kidney disease, 26% had diabetes, 14% patients had prior myocardial infarction, heart failure was found in 11%, and 10% had a history of stroke (13); all these comorbidities were more severe in patients who had higher level of serum uric acid (hyperuricemia) (14). It is believed that, on average, the patients with chronic gout have at least four comorbidities, while this number may increase up to seven or more in 10% patients (15, 16). The exact link between gout and extraarticular comorbidities is still not completely understood (17). The patients with subcutaneous tophi have higher chances of comorbidities (18).

### Diagnosis and Role of DECT

Gout is often clinically suspected based on the classic presentation, demographics, and associated risk factors. Gold standard diagnostic test for confirmation of gout is the demonstration of birefringent MSU crystals under polarized microscopy in synovial fluid obtained by joint aspiration in patients with acute flare of gout (19, 20). Gout can often be diagnosed on clinical findings alone in primary care settings (21). For patients with an intermediate risk in whom arthrocentesis is not readily available, additional non-invasive radiological evaluation may help further support the diagnosis. Although plain radiography is one of the initial radiological tests typically sought in such patients and is often used for confirmatory evidence, the sensitivity for the detection of early gout is limited at ~30% (22). For this reason, DECT has emerged as an invaluable alternative for the accurate and efficient depiction of urate crystal in patients presenting with clinical signs and symptoms of gout (23–25). DECT has previously been used as a highly sensitive and specific diagnostic tool for diagnosis of gout by accurately depicting urate crystal deposition within the affected joints (26–28).

DECT utilizes relative absorption of X-rays at different energy levels (usually at 80 and 140 kVp) to differentiate between different materials based on the principle of difference in their atomic number (29). The higher atomic number material like calcium will show higher difference in attenuation when imaged with X-rays of two different energy levels due to photoelectric effect in contrast to the low atomic number material like MSU crystals, which does not demonstrate significant difference in attenuation. The ability to differentiate two materials is therefore directly proportional to the atomic number and electron density of the material (30). By using independent tube current modulation, low electronic noise detectors, and tin filtering of the higher kilovoltage (kV) tube's spectra, DECT produce high-resolution images with excellent energy spectral separation and helps in keeping the radiation dose of DECT comparable to conventional monoenergetic CT (31). Two material decomposition algorithm is used to differentiate MSU crystals from the calcium deposits. Although on single energy level CT, both the calcium and MSU crystal deposits appear bright, but these materials can be differentiated on DECT and color coded using a special software (green used for MSU deposits and blue for calcium deposits). Currently, five types of DECT scanner are available including dual source DECT, twin-beam single-source CT with gold filter, rapid kilovoltage-switching source with gemstone scintillator detector (GSI), dual-layer multidetector DECT, and dual-scan single source (29).

One of the limitations of DECT as compared to plain radiography, ultrasonography, and MRI is ionizing radiation, which can be of concern particularly in cases that require repeated imaging on follow-up or imaging of all the joints to see involvement of individual joint. The radiation dose is, however, similar to conventional CT (usually <1 mSv) (24).

### Detection of MSU Crystals in the Cardiovascular System

**Figure 1:**
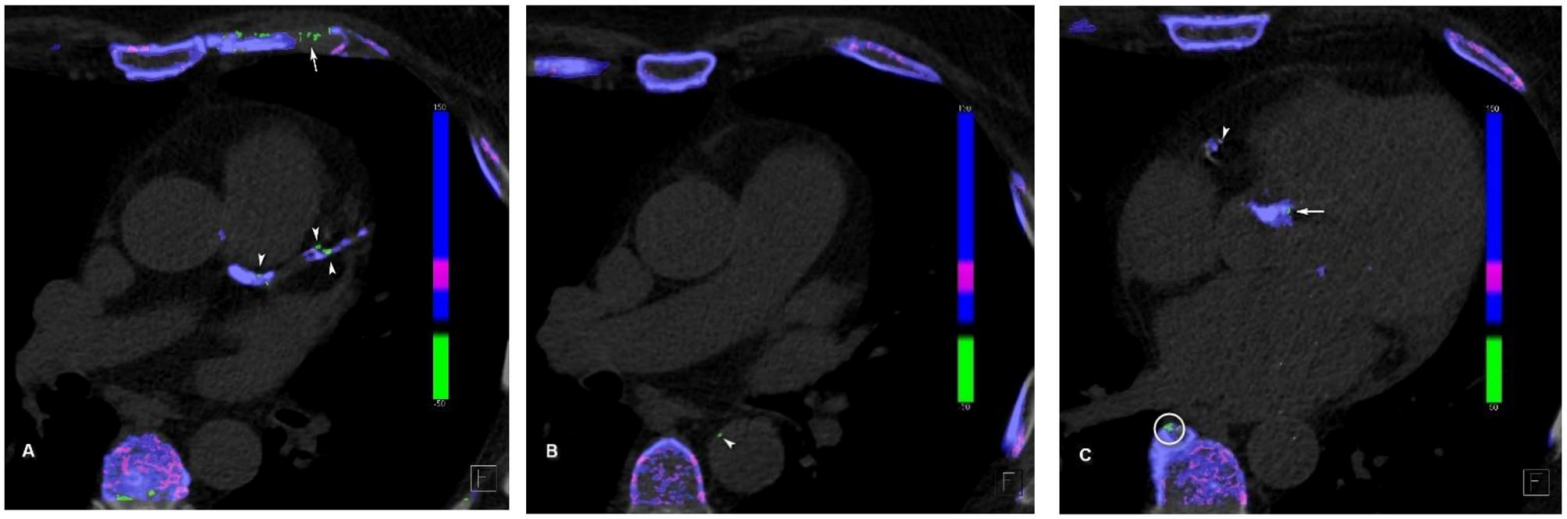
Cardiovascular urate deposits. **(A)** Urate crystal (green) deposits in the left main coronary artery, left anterior descending artery, and its first diagonal branch (arrowheads). Note the artifactual green deposits in the coastal cartilage (arrow), as it has similar attenuation profile as uric acid. **(B)** Mural urate deposit in the descending thoracic aorta (arrowhead). **(C)** Urate deposit in the right coronary artery (arrowhead) and in the aortic valve calcification (arrow). Note the artifactual green deposit at the edge of vertebral osteophyte (circle).

MSU deposits were thought to be artifactual earlier, as identified on DECT in extraarticular regions especially in the vessels. Mallinson et al. (32) showed the MSU deposits as an interesting artifact in four cases in their study with calcified plaques. However, later, it was found that MSU crystals are deposited in the vasculature of the patients with high serum uric acid level (Figure 1), and this resulted in activation of the inflammatory response in the vessel wall.

MSU crystal activates the NLRP3 inflammasome once they are engulfed by the macrophages (33), resulting in the generation of proinflammatory cytokines, which leads to atherosclerotic cascade activation and progression (34) and hence increased risk of cardiovascular events in such individuals. Second possibility is related to increasing smooth muscle cell proliferation in the vessels following oxidative stress secondary to high uric acid levels (35), which in return causes atherosclerotic disease progression and thereby increased cardiovascular risk (36).

There are several studies that have documented the association of hypertension, cardiovascular events, and stroke with gout (37–39). Autopsies have confirmed MSU deposits in the myocardium and endocardium on histopathology as evident by the tophi within the myocardium with involvement of the epicardial fat as well, as found in one of the autopsies (40). Involvement of the conduction pathways was also reported in one of the autopsies specimen of the individual who had complete heart block (41). It has been reported that patients with known gout presented with acute myocarditis. The histopathology specimen confirmed the presence of MSU deposits admixed with inflammatory cells.

A study done by Barazani et al. (42) quantified the uric acid in the vasculature of the gout patients using DECT. They included 31 gout patients along with 18 controls who underwent DECT of the chest and abdomen. Using the DECT software algorithm, MSU crystals were differentiated from calcification within the plaques involving the aorta, coronaries, and iliacs. The patients included in this study had different comorbidities; 39% had history of diabetes, 22% had cardiovascular disease, and 48% had history of smoking. MSU crystal deposition was found in significant volume involving the aorta in patients who had prior history of gout (median, 43.9 and 2.9 for gout and control groups, respectively, with *P-*value of 0.01). When considered separately, the number of green spots (MSU deposits) were also found to be higher in the gout patients relative to the second group (median of 20 and 1.5 for gout and control groups, respectively, with *P*-value of 0.008). The difference was, however, found insignificant among the two groups regarding MSU deposition and green spots when other factors were considered including age, gender, known DM, and cardiovascular disorders, *P* = 0.53 and *P* = 0.39, respectively. MSU deposition was found to be higher with increasing age (*P* = 0.08) with relatively larger size of the deposits in the older age group. MSU deposits found in the coronaries and iliac arteries were also found significantly larger in gout subjects. Fifty-five percent of the gout patients were found to have MSU deposits in the coronaries (*P* < 0.001) and 54% in the iliac arteries (*P* = 0.005), while the control group had 0 and 11%, respectively. Similarly, evidence of urate crystals in the aorta was found to be greater in the gout group as compared to the control group. There were certain limitations of this study including the small sample size, inability to confirm the presence of MSU crystals histologically, demographically unbalanced sample, and the findings were not correlated with serum biochemical markers in the gout and control groups.

Another study done by Abdellatif et al. (43) demonstrated the presence of MSU crystals in coronary arteries in 84.62% of the patients with tophaceous gout, while only one subject (2.08%) in the control group had coronary MSU deposits. The sensitivity of DECT was 84.62% with specificity of 97.92% in patients within the same age and gender groups.

Klauser et al. (5) demonstrated the detection of MSU crystals in gout patients. They selected 59 patients with gout with high serum uric acid level over 15 years, having average serum uric acid level of more than 6.35 mg/dl when they were imaged, with a mean of two gout attacks per year. A total of 55 subjects (93.2%) had calcific coronary atherosclerotic disease with mean calcium score of 900 AU. It was found in over 80% of the patients that MSU crystal deposits were present in the vasculature; these patients had mean duration of gout of 3 years, with mean serum uric acid level above 7.4 mg/dl when they were imaged. 16.9% had deposits in the aorta, and 32.2% were found to have urate crystal deposition in the coronary arteries, while 37.3% of the patients had deposits at both sites. Only eight (13.6%) gout subjects were negative for vascular MSU deposits in contrast to the 85.1% in the control group. Out of the 47 subjects in the control group, only seven were found to have vascular urate crystal deposits (mean serum uric acid level of 6.8 mg/dl), and none had history of prior gout. Of the control group who were found to have vascular deposits of MSU, 4.3% had involvement of the aorta, 4.3% had involvement of the coronaries, and 6.4% demonstrated involvement of both sites. The vascular urate deposits in the control group including the involvement of the coronaries were found to be relatively lower. In terms of involvement of the mitral or tricuspid valves, the difference was small among the two groups. Similarly, the coronary calcium score was also found to be higher in the gout individuals as compared to the control group (*P* = 0.001). The correlation between serum uric acid levels and vascular MSU deposits was, however, not found significant.

This study also included imaging of six fresh cadavers (mean age at death, 79 years); the prior history of gout or hyperuricemia was not known in these cadavers. Three showed MSU crystal deposition involving the thoracic aorta, coronaries, and combination of aortic and coronaries involvement with MSU deposits on mitral valve as well. These MSU deposits were confirmed on histopathology as well in seven out of eight specimens using polarized light microscopy, confirming the positive predictive value of DECT to be 87.5%.

Disveld et al. (44) showed a strong association between higher prevalence of CVS diseases in patients who had gout. Pagidipati et al. (45) found in their study that in patients with acute coronary syndrome, there was an association between increased risk of cardiovascular events and increased serum uric acid levels, even without established diagnosis of gout. Pascart et al. (46), however, found that there was no association between higher risk of cardiovascular events and volume of MSU deposition in knees and feet.

Andres et al. (10) found an association between higher coronary calcification and MSU crystal deposits involving the knees and first metatarsophalangeal joint in individuals who had higher serum uric acid levels; this study was limited though because these calcifications were not histpathologically confirmed.

Several studies have also confirmed MSU deposits involving the cardiac valves that were confirmed histopathologically and/or using echocardiograms (47, 48). Different studies have shown the involvement of all the cardiac valves including mitral, tricuspid, aortic, and pulmonary valves (40, 49, 50). Postsurgical specimen following carotid endarterectomy and aortic aneurysm also confirmed the MSU deposits adjacent to the usual cholesterol containing plaques (51, 52).

### Uric Acid Urolithiasis

Nephrolithiasis is a common and costly condition, with reported increasing incidence and prevalence ranging from 4 to 20% in developed countries (53–56). Several risk factors are reported for urinary calculi resulting in varied composition of the renal calculi. The most common compositions for urinary tract calculi include mixed calcium oxalate and phosphate, pure calcium phosphate, struvite (triple phosphate), and uric acid. The relative incidence of uric acid stones varies widely ranging from 8 to 10% of the urinary calculi in the United States to up to 28% in Pakistan (57). There are many etiological causes for the formation of uric acid calculi, and these include multiple acquired and genetic factors such as dehydration, metabolic syndrome, diarrheal states, hyperuricosuria, and low urinary pH and its many causative factors, including gout (58, 59). It is reported that 10–20% of the gout patients have urate nephrolithiasis (60, 61).

Imaging is a fundamental component of the investigation and planning of the management of the urinary tract calculi. Among the imaging methods, computed tomography (CT) is the gold standard, with sensitivity and specificity of 95% when considering non-enhanced scans. Other than localizing and measuring urinary tract calculi, CT can provide additional information that guides treatment such as estimating stone composition. Even though there is considerable overlap in composition among different types of calculi, non-contrast CT can provide some information and help in differentiation according to the attenuation. Uric acid stones attenuation is more often between 200 and 400 HU, while calcium oxalate is more likely range from 600 to 1200 HU (62).

**Figure 2:**
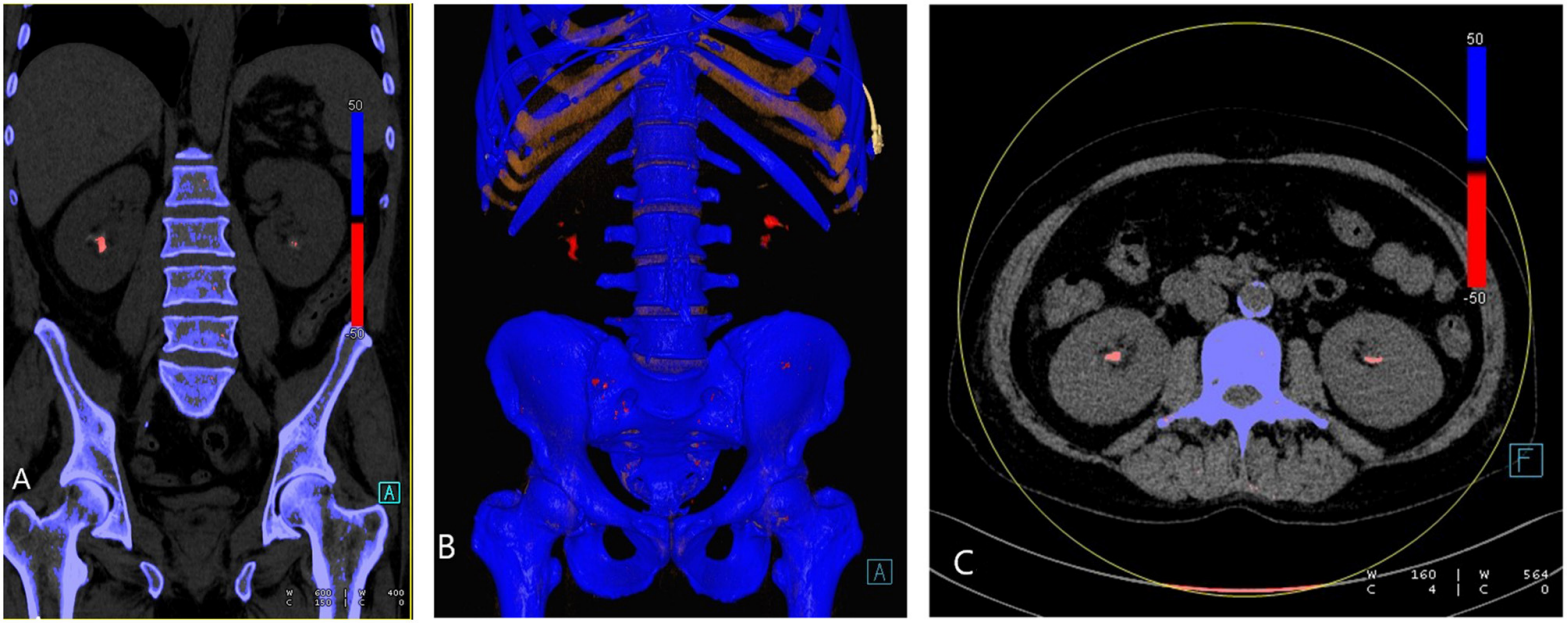
Urate renal stones. **(A)** Coronal reformatted, **(B)** 3D rendered, and **(C)** axial images demonstrating bilateral renal urate stones (red).

The advent of dual-energy CT has allowed for new possibilities in the evaluation of the composition of urinary tract calculi. Based on the differences in attenuation, the selected materials at different energy levels, and because of their different atomic numbers, DECT can detect stone composition. Uric acid calculi are composed primarily of low atomic number elements such as nitrogen, carbon, hydrogen, and oxygen. Non-urate containing calculi, on the other hand, are primarily composed of elements with higher atomic numbers such as calcium, phosphorus, and sulfur. DECT software algorithm color codes the calculi, conventionally depicting uric acid calculi (Figure 2) as red and calcium calculi as blue (63, 64). A recent systematic review and metanalysis by McGrath et al. has demonstrated that DECT has a specificity of 88% and sensitivity of 98% for uric acid dominant stones, making DECT a relatively accurate imaging tool to determine the composition of urinary calculi *in vivo*, which allows for a more prompt management planning and earlier non-invasive urine alkalinization therapy for the uric acid calculi, potentially reducing health care costs (65).

### MSU Deposition in Spine

**Figure 3:**
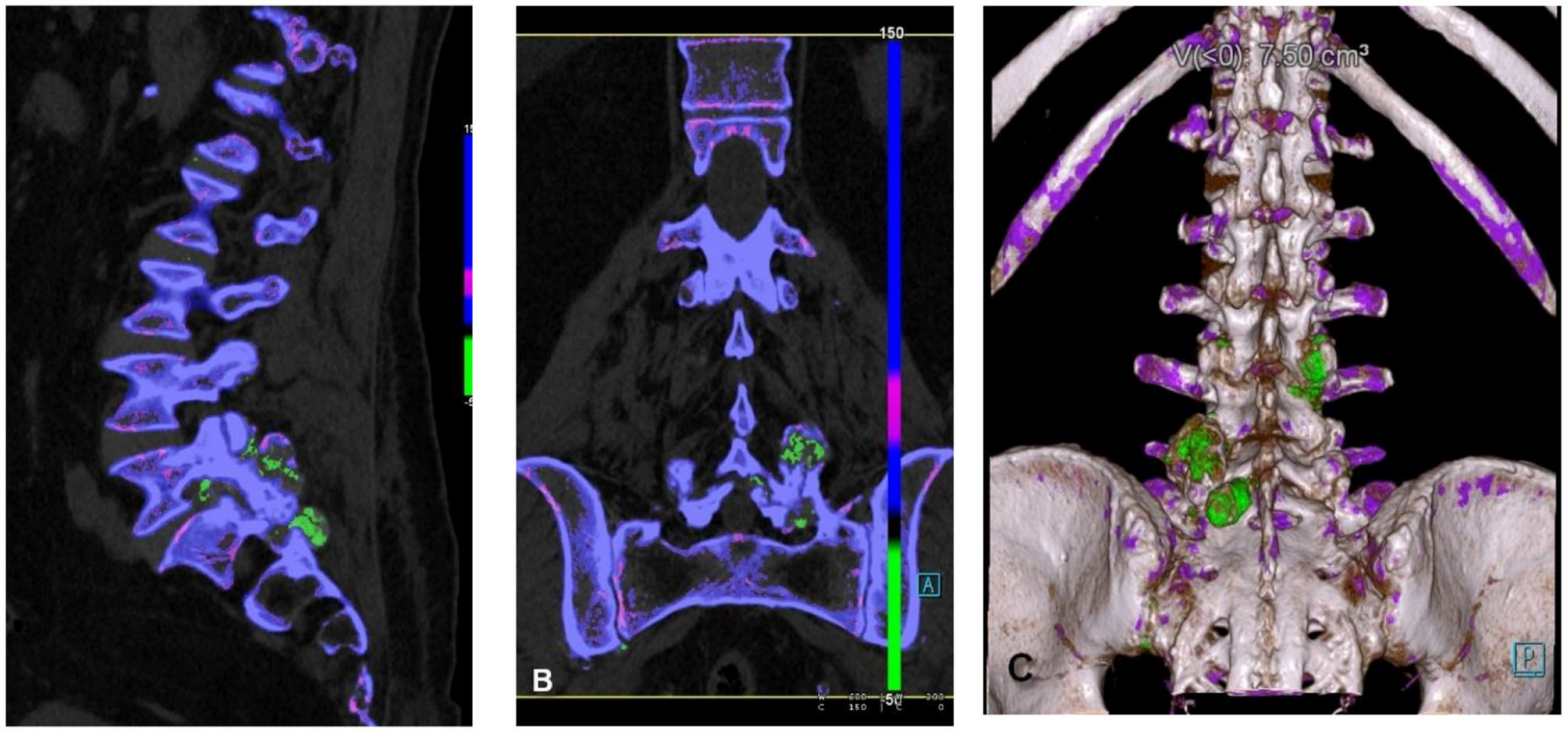
Spinal urate deposits. **(A)** Sagittal reformatted, **(B)** coronary reformatted, and **(C)** 3D-rendered images demonstrating urate deposits in facet joints (green).

MSU deposition in the spine has been reported in different studies (the first case ever detected was in 1950) (66) with the facet joints of the lumbar spine being the most common site of involvement (67, 68), although MSU deposits have been reported elsewhere as well (67) (Figure 3). Clinically, symptoms may mimic degenerative disk disease and/or infection due to compression of nerve roots or spinal cord itself by the tophi. Application of DECT imaging may avoid surgery and hospitalization (68, 69) in gout patients with symptomatic spinal manifestation. Chotard et al. showed in their retrospective study on gout subjects that 60% DECT of these subjects had urate deposits in the spine out of which 83% were symptomatic (70). A study done by Anastasia S et al. found that gout patients had higher rate and severity of back pain as compared to control subjects. The gout subjects were found to have MSU deposits, which were in proportion to the level of serum uric acid (71). It has been reported that symptoms resolved after urate lowering therapies/medical management in patients who presented with back pain and were known to have gout (69, 72).

### Artifacts

It is important to know the common artifacts arising in DECT gout assessment, so that they are not misinterpreted as tophi. Several types of MSU deposit mimics have been identified in prior researches. Some factors causing artifacts include beam-hardened artifacts due to metals, motion artifact, image noise at low kilovoltage peak (kVp), and thick callous skin such as in nail beds and heel. Furthermore, in middle-aged population, costal cartilage and intervertebral disks mimic similar density to that of urate crystals, leading to artifactual green deposits on DECT (32, 73, 74).

It is interesting and intriguing to reiterate here that previous vascular MSU deposits were considered artifactual as well.

## Conclusion

With the advent of DECT, it has come to light that MSU crystal deposition is not confined to joints or periarticular soft tissues but can be visualized in various extraarticular sites as well other than known conventional sites including cardiovascular, renal, and spine, illustrating the systemic nature of the disease. This will help us in future research for *in vivo* detection of MSU crystals in extraarticular sites with more confidence and will help physicians to increase their awareness of the presence of MSU deposits in multiple systems likely resulting in various comorbidities in gout patients. Future studies can be done using DECT to determine the frequency of MSU depositions in other systems as well, which can be helpful in the early detection and screening of the gout patients.

## Author Contributions

MA, SM, and DF completed the research and manuscript writing. SM also prepared medical images. SN was involved in editing and provided direction. All authors contributed to the article and approved the submitted version.

## Conflict of Interest

SN has a Master Research Agreement between the University of British Columbia and Siemens Healthcare. The remaining authors declare that the research was conducted in the absence of any commercial or financial relationships that could be construed as a potential conflict of interest.
